# Microwave-assisted synthesis of silica quantum dots: a novel approach for targeting PI3K/AKT signaling in breast cancer therapy

**DOI:** 10.1039/d5ra04715c

**Published:** 2025-10-20

**Authors:** Anitha Selvaraj, Kumar Ponnuchamy

**Affiliations:** a Food Chemistry and Cancer Biology Lab, Department of Animal Health and Management, Alagappa University Karaikudi-630 003 Tamil Nadu India kumarp@alagappauniversity.ac.in

## Abstract

In this study, a facile and efficient approach was adopted for the first time to synthesize silica quantum dots (SiQDs) using tetraethyl orthosilicate (TEOS) as a precursor and ascorbic acid as a reducing agent. The synthesis was achieved *via* microwave-assisted hydrolysis of TEOS in the presence of ascorbic acid, resulting in the formation of stable, highly fluorescent SiQDs. Extensive high-throughput characterization techniques were employed to investigate the optical, morphological, and structural properties of the as-prepared SiQDs, confirming their uniform size distribution, strong photoluminescence, and moderate stability. The therapeutic potential of SiQDs was explored against breast cancer cell lines (MCF-7 and MDA-MB-231), where they exhibited significant cytotoxicity and selective anticancer activity. Mechanistic investigations revealed that SiQDs effectively induce apoptosis in cancer cells by downregulating the PI3K/AKT signaling pathway, a critical regulator of cell survival and proliferation. These findings underscore the potential of SiQDs as promising anticancer agents in precision cancer therapy, offering a novel strategy for targeted cancer treatment. The study also paves the way for the design and development of silica-based nanomaterials with tailored properties for biomedical applications.

## Abstract 2

Silica quantum dots (SiQDs) targeting PI3K/AKT signaling of breast cancer.

## Graphical abstract

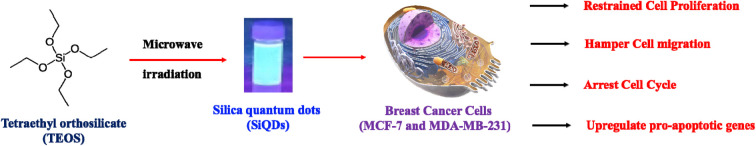

## 1 Introduction

Today, cancer is a major global public health issue that is devoid of effective diagnosis and treatment.^1–3^ Most often, chemotherapeutic agents (such as doxorubicin and cisplatin) are non-specific, which eventually have no ability to differentiate cancer and normal cells, causing adverse side effects that limit therapeutic outcomes.^4,5^ In view of this, minute theranostic particles called quantum dots (QDs) have emerged as a competitive agent among the existing strategies, owing to their myriad of fascinating features, including photostability and strong fluorescence, offering cutting-edge applications in the field of biomedical science.^6–8^

Despite the fact that QDs are versatile agents that often employ heavy metals (*e.g.*, cadmium), which raise concerns due to their inherent toxicity and environmental risks, thereby impeding their broader use in clinical applications.^9–12^ For example, cadmium-containing QDs upon exposure pose serious reproductive toxicity, including impaired fertility and multigeneration effects.^13^ In order to alleviate this, surface modification and dissolution are required to reduce toxicity and enhance biocompatibility.^14,15^ To address the above limitation, researchers show considerable interest in developing heavy-metal-free QDs.^16,17^

In particular, silica-based nanomaterials stand out as unique due to their chemical inertness and outstanding biocompatibility.^18,19^ Meanwhile, pure silica is non-luminous, necessitating the incorporation of functionalized agents to achieve photoluminescence. Most often, classical Stöber methods involve tetraethyl orthosilicate (TEOS) or 3-aminopropyltriethoxysilane (APTES) to yield silica nano structures, which require the use of expensive and potentially hazardous reducing agents.^20,21^ In addition, synthetic methods for developing silica-based nanomaterials often involve extensive reaction times, high temperatures, or hazardous reagents, which limit scalability and practical application.^22^

In this journey, silica quantum dots (SiQDs) are a versatile agent that demonstrates promising capabilities in bioimaging and drug delivery across multiple studies. In 2008, Fujioka *et al.* developed passively oxidized SiQDs as bioimaging probes, showcasing mitochondrial activity and lactate dehydrogenase (LDH) release in cultured HeLa cells.^23^ Later, Erogbogbo *et al.* (2008) reported the synthesis of SiQDs using phospholipid micelles and demonstrated their imaging capabilities in pancreatic cancer cells using confocal microscopy.^24^

Beyond basic imaging, Erogbogbo *et al.* (2011) developed a folate- and antimesothelin-conjugated SiQDs, evaluating its selective uptake into pancreatic cells for cancer imaging applications.^25^ Similarly, Handa *et al.* (2013) explored the efficacy of drug-modified SiQDs (alminoprofen) targeting hepatocarcinoma (HepG2) cells.^26^ Tu *et al.* (2016) highlighted the use of fluorescent SiQDs for immunostaining liver cancer cells (SKOV3 and CHO), demonstrating comparable performance to fluorescein isothiocyanate (FITC).^27^ In an entirely different direction, Chen *et al.* 2020 reported the synthesis of fluorescent N-doped silica QDs derived from wheat straw and ionic liquids, which showed potential.^28^

Most of the research involving SiQDs focuses on imaging and sensing. However, targeting cancer-specific signaling remains largely unexplored. Notably, the PI3K/AKT pathway is a pivotal regulator of cell growth, survival, metabolism, and migration; its dysregulation is a hallmark of many cancers, particularly breast cancer.^29^ PI3K mutations and PTEN loss often drive tumor proliferation and therapy resistance, highlighting the urgent need for agents that can interfere with this signaling pathway.^30^

Keeping this in mind, the present study introduces a novel microwave-assisted synthesis of fluorescent SiQDs using TEOS and ascorbic acid, which benefits from the efficiency, uniform reaction conditions, and reduced synthesis times afforded by microwave heating. While both TEOS and ascorbic acid have been individually utilized in nanoparticle synthesis, the combination of these reagents in a microwave-assisted, single-step protocol offers a rapid and biocompatible approach to producing fluorescent SiQDs.

Unlike traditional multi-step or metal-dependent methods, this route utilizes only benign and accessible reagents, potentially reducing environmental and biological toxicity. This approach utilizes the formation of a biocompatible silica derived from TEOS and generates luminescent QDs in the presence of ascorbic acid through a rapid, scalable, and environmentally friendly process.

The resulting SiQDs are characterized and evaluated for their anti-tumor activity in breast cancer cell lines (MCF-7 and MDA-MB-231), focusing specifically on their ability to inhibit PI3K/AKT signaling and suppress cancer cell proliferation. To the best of our knowledge, this represents the first use of SiQDs as direct modulators of a cancer-critical pathway, suggesting their potential as next-generation nanotheranostic agents for targeted breast cancer therapy.

## 2 Experimental section

### 2.1 Chemicals and materials

Sigma Aldrich Chemicals Private Limited, Bangalore, India provided tetraethyl orthosilicate (TEOS) and ascorbic acid (AA). Cell culture-based chemicals such as Dulbecco's Modified Eagle Medium (DMEM), Fetal bovine serum (FBS), and antibiotics (streptomycin/penicillin) were procured from HiMedia Laboratories Private Limited, Thane, India. Primers for gene expression studies were obtained from Eurofins Genomics India Private Limited, Bangalore, India. Ultrapure water from a Milli-Q Integral 5 system (Millipore, USA) was used to rinse all glassware thrice before use and to prepare all aqueous solutions.

### 2.2 Cell culture

Human breast cancer (MCF-7 and MDA-MB-231) and normal (HEK-293) cell lines were received from the National Centre for Cell Science, Pune, India and cultured in DMEM supplemented with FBS and antibiotics.

### 2.3 Synthesis of silica quantum dots (SiQDs)

In the present study, SiQDs were synthesized *via* a straightforward microwave irradiation technique. Briefly, 2 mL of tetraethyl orthosilicate (TEOS) is dissolved in 8 mL of Milli-Q water. To this mixture, about 2.5 mL of freshly prepared l-ascorbic acid (0.1 M) is added and stirred for 10 min to achieve uniform homogeneity. The as-prepared mixture was subjected to microwave irradiation at 160 °C for 10 min using a jar. After irradiation, the residual impurities were removed by using a dialysis membrane with a molecular weight cut-off of 1 kDa. The final product was lyophilized and used for further characterization.

### 2.4 Instrumentation

High-throughput analysis, such as absorption (UV-visible spectrophotometer, Evolution-201, Thermo, USA), photoluminescence (photoluminescence spectrophotometer, Optistat, Oxford Instruments; Varian Cary Eclipse, USA), functional group analysis (Fourier-transform infrared spectroscopy, Nicolet iS5 FTIR spectrometer, Thermo, USA), morphology (high-resolution transmission electron microscopy, HR-TEM, coupled with selected area electron diffraction, SAED, JEOL JEM-2100 PLUS, Japan), diffraction patterns (X-ray diffractometer, XRD, X'Pert Pro, PANalytical, UK), elemental composition (energy-dispersive X-ray spectroscopy, EDS, TESCAN Oxford Instruments, Czech Republic; and X-ray photoelectron spectroscopy, XPS, PHI-VERSAPROBE III XPS system, USA), and zeta potential measurements (Zetasizer Nano-ZS90, Malvern, UK) were used to characterize SiQDs.

### 2.5 Cytotoxicity and apoptotic staining methods

MTT assay is performed to ascertain the effect of SiQDs (0–200 μg mL^−1^) on breast cancer (MCF-7 and MDA-MB-231) cells.^31^ The biocompatibility of SiQDs was investigated using human embryonic kidney (HEK-293) cells. For the MTT assay, cisplatin was used as a positive control, and the selective index (SI) was calculated.^32^ Clonogenic assay is performed utilizing SiQDs to address the colony-forming ability of breast cancer (MCF-7 and MDA-MB-231) cells. The characteristic features of apoptosis in breast cancer cells upon treatment with SiQDs were evaluated by using dual (involving acridine orange/ethidium bromide, AO/EtBr), nuclear (Hoechst 33342), generation of reactive oxygen species (ROS, DCFH-DA), and mitochondrial membrane potential damage (rhodamine 123) based on fluorescence staining. An Accu-Scope (EXI-300, USA) fluorescence microscope was utilized for recording images at 20× magnification.

### 2.6 Cell migration assay

A scratch assay was performed to investigate the ability of cell migration in breast cancer cells (MCF-7 and MDA-MB-231) by creating a scratch in a six-well plate.^33^ The cells were treated with IC_50_ value of SiQDs followed by incubation (24–48 h). At specified intervals, an Accu-Scope (EXI-310, USA) inverted microscope was utilized to record the wound closure at 4× magnification.

### 2.7 Cell cycle analysis

Cells were treated with IC_50_ concentrations of SiQDs for 24 h.^34^ After treatment, cells were trypsinized, washed (with PBS thrice), fixed (70% ethanol), and stored (−20 °C) overnight. The fixed cells were further PBS-washed, incubated with RNase A (at 37 °C for 1 h), and stained with propidium iodide (PI) for 15 min at room temperature. The cell cycle phase analysis was carried out using FACS Fortessa X-20 (Becton, Dickinson and Company (BD), USA) and analyzed using FlowJo software, USA.

### 2.8 Gene expression

Total RNA from IC_50_-treated breast cancer (MCF-7 and MDA-MB-231) cells was isolated using TRIzol™ reagent.^35^ The total RNA was quantified (Quantus Fluorometer, Promega, USA), and cDNA was constructed (PrimeScript 1st strand cDNA Synthesis Kit one-step RT-PCR, Takara, Japan). Gene expression studies were carried out using semi-quantitative RT-PCR. The primers used in the study were tabulated in SI Table 1. The PCR product was subjected to amplification by agarose gel electrophoresis and viewed by a bioimaging system (GE ImageQuant LAS-500, USA).

### 2.9 Statistical analysis

GraphPad Prism V.9.3 statistical software was used to ascertain the mean ± standard error (SE) of three independent experiments. A Student's *t*-test was considered to calculate the difference in means, with *p* < 0.05 considered statistically significant. Statistical significance was annotated as follows: *p < 0.05, **p < 0.01, ***p < 0.001, and ****p < 0.0001.

## 3 Results and discussion

**Fig. 1:**
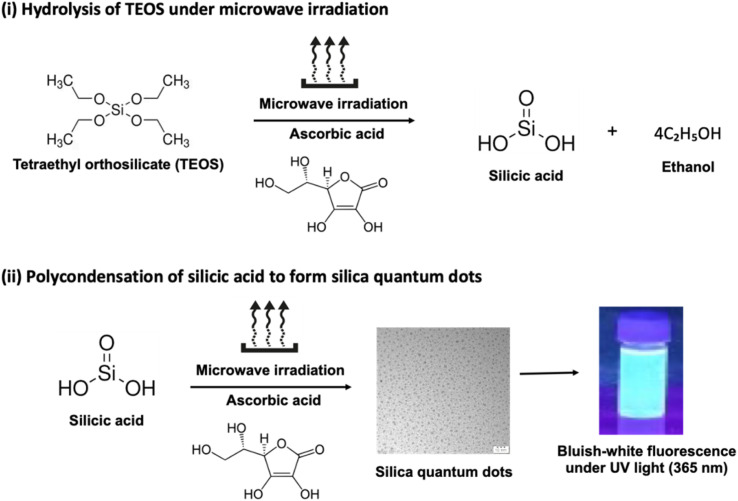
Schematic illustration involved in the synthesis of silica quantum dots (SiQDs).

In the present study, tetraethyl orthosilicate (TEOS) was reacted with ascorbic acid under microwave irradiation, resulting in the formation of silica quantum dots (SiQDs). The process begins with the hydrolysis of TEOS, producing silicic acid (Si(OH)_4_) as an intermediate (Fig. 1). Notably, silicon remains in the +4 oxidation state throughout this transformation, and no redox chemistry is involved. Under rapid and homogeneous heating provided by microwave irradiation, Si(OH)_4_ undergoes condensation, leading to the nucleation and growth of silica nanostructures. In this pathway, ascorbic acid plays a crucial role as a surface passivating agent, stabilizing the nascent quantum-sized particles and minimizing agglomeration, thereby yielding stable SiQDs (Fig. 1).

**Fig. 2:**
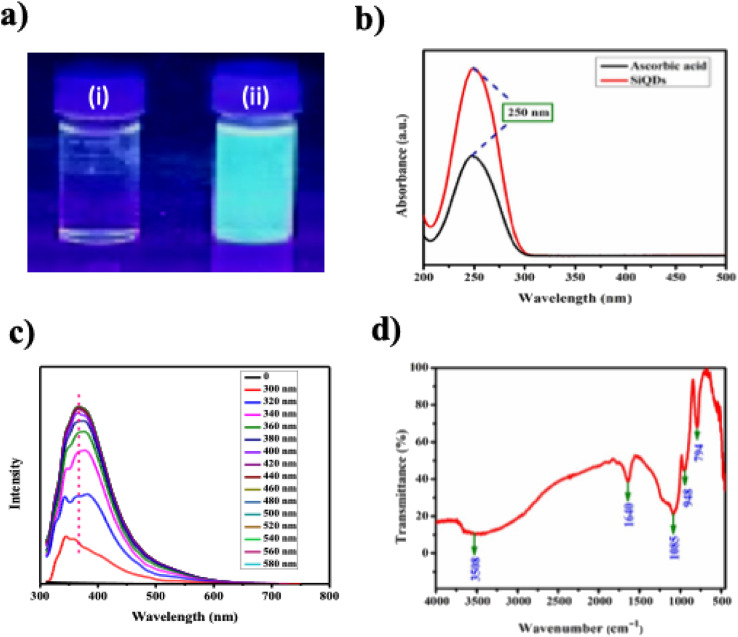
(a) Visual observation of color change under ambient (i) and UV light at 365 nm (ii); (b) UV-vis absorption spectrum indicating the optical properties; (c) photoluminescence (PL) spectrum showing emission characteristics; (d) FTIR spectrum confirming functional groups and surface chemistry.

As shown in Fig. 2a, SiQDs exhibited strong bluish-white fluorescence under UV light (365 nm).^37^ A strong and stable fluorescence emission is noted from SiQDs, typically due to minimal self-quenching and aggregation caused by the surface passivating agent, ascorbic acid.^36^ UV-vis absorption peak at 251 nm, attributed to the electronic transition of SiQDs, reflecting its quantum-sized nature (Fig. 2b).^38^ Moreover, the use of microwave irradiation ensures uniform particle size and improved optical properties.^39^

Fig. 2c shows the fluorescence spectra revealing an excitation-dependent emission by SiQDs.^40^ It has been noted that excitation-dependent emission is a hallmark of QDs, reflecting their unique optical properties.^41,42^ The emission peaks shift from blue to green with increasing excitation wavelength (300–400 nm) as shown in SI, Table 2. This behavior arises from surface defect states and size variations within the QDs, highlighting their tunable optical properties suitable for sensing and bioimaging applications.^43^

Distinct chemical signatures for ascorbic acid, TEOS, and the synthesized SiQDs were discerned in FTIR spectra. Ascorbic acid showed strong O–H stretching bands (3313–3524 cm^−1^), a prominent C=O stretch at 1751 cm^−1^, and C–O vibrations, consistent with its polyhydroxy-lactone structure (SI Fig. 1 and SI Table 3).^44^ TEOS exhibited characteristic C–H stretching (2975–2981 cm^−1^), Si–O–C asymmetric stretching (1070–1165 cm^−1^), and Si–O–Si symmetric stretching at 785 cm^−1^, indicative of its alkoxysilane framework (SI Fig. 2 and SI Table 4).^45^ The SiQDs spectrum showed the disappearance of C=O and Si–O–C bands, and the emergence of strong Si–O–Si (1085, 792 cm^−1^) and Si–OH (946 cm^−1^) peaks, confirming successful hydrolysis and condensation of TEOS (Fig. 2d and SI Table 5). A broad O–H peak at 3508 cm^−1^ suggests surface hydroxylation, improving dispersibility. The reduction in organic-related peaks and formation of siloxane bonds imply that ascorbic acid played a dual role as a reducing and stabilizing agent during microwave-assisted synthesis, enabling the formation of stable, functionalized SiQDs (SI, Table 6). The results from the study is consistent with earlier reports during the formation of silica-based nanomaterials.^46,47^

**Fig. 3:**
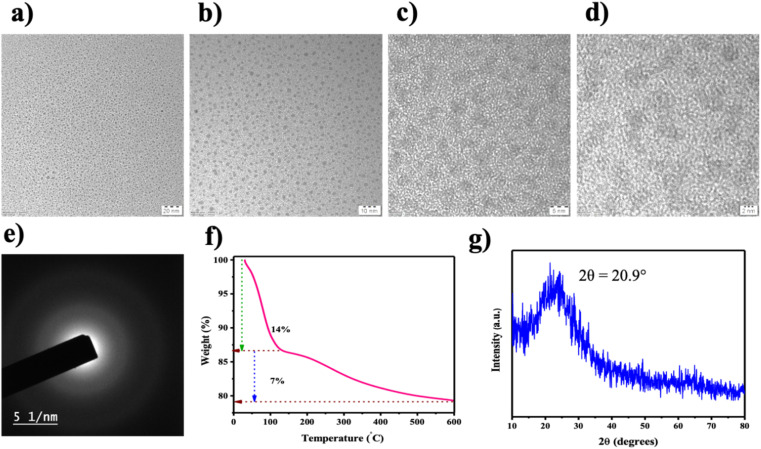
SiQDs: (a–d) High-Resolution Transmission Electron Microscope (TEM) images depicting morphology at different magnifications; (e) Selected Area Electron Diffraction (SAED) pattern; (f) thermogravimetric analysis; (g) X-ray diffraction (XRD) pattern revealing the crystalline structure.

TEM analysis showed the occurrence of spherical-shaped SiQDs, predominantly between 4–6 nm in diameter (Fig. 3a–c). Further, the micrographs showed no distinct lattice fringes, confirming that the SiQDs are predominantly amorphous.^48^ Interestingly, the minor crystalline domains observed in HR-TEM may indicate the presence of residual components, possibly originating from the synthesis process or precursor materials (Fig. 3d). The average particle size distribution of the synthesized SiQDs was found to be approximately 6 nm, indicating a narrow size distribution and is consistent with quantum confinement behavior (SI Fig. 3). The SAED pattern of the SiQDs showed diffuse rings rather than sharp diffraction spots, confirming the amorphous nature of the silica shell^49^ (Fig. 3e).

Further, thermogravimetric analysis was executed, wherein the stability of these SiQDs was studied with respect to temperature (Fig. 3f). A significant change in weight, composition, and stability of these structures happens when the temperature exceeds 200 °C.^50^ However, the stability of these QDs was prevalent till 100 °C. XRD patterns of SiQDs exhibit a broad peak indicative of their amorphous nature, centered at 2*θ* = 20.9° with a characteristic of a disordered Si–O–Si network (Fig. 3g).

**Fig. 4:**
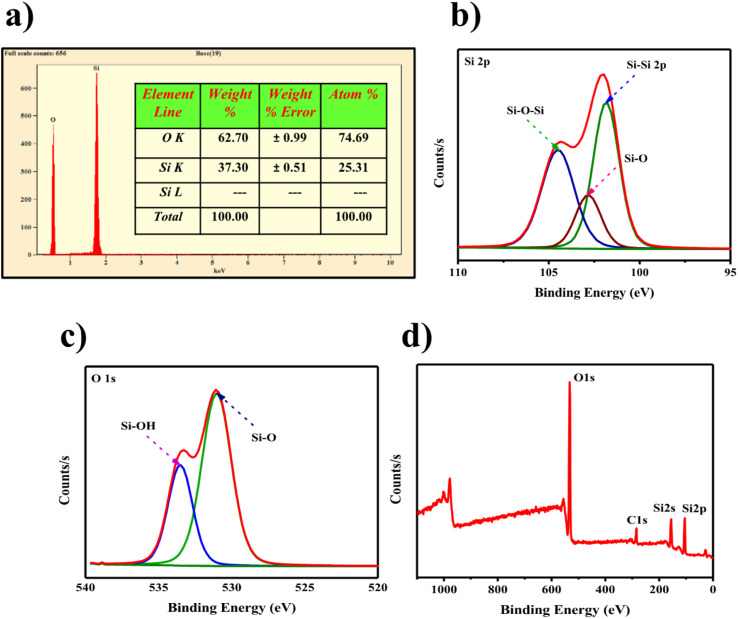
SiQDs. (a) Energy Dispersive X-ray Analysis (EDAX) for elemental composition; (b–d) X-ray Photoelectron Spectroscopy (XPS) spectra illustrating surface chemical states and bonding environment.

Energy dispersive X-ray (EDAX) revealed the presence of Si, O, and H elements in these SiQDs^51^ (Fig. 4a). The X-ray photoelectron spectroscopy (XPS) portrayed the presence of conformational states of these atoms at the elemental state, along with surface chemistry properties of SiQDs (Fig. 4b–d). In this study, predominant peaks owing to the 530 eV and 537 eV indicate the presence of Si–O and Si–OH groups. The peaks at 102, 104, and 106 eV correspond to the presence of Si_2_ps, Si–O, and Si–O–Si, respectively, indicating the presence of oxygen-containing moieties due to the interaction with water molecules.^52,53^

Dynamic light scattering (DLS) analysis revealed that the synthesized SiQDs possess a multimodal size distribution, with the smallest peak centered at 2.28 nm (9.6% intensity), indicating the presence of well-dispersed, discrete QDs (SI Fig. 4). In contrast, two predominant peaks at 231.6 nm and 5411 nm are suggestive of particle aggregation or incomplete dispersion, possibly due to surface interactions among SiQDs during measurement. The relatively high polydispersity index (PDI = 0.885) and Z-average diameter (1190 nm) further confirm the heterogeneous nature of the sample. These findings are consistent with previous reports on size variation in colloidal SiQDs under similar conditions.^54^ Additionally, the measured zeta potential of −7.91 mV reflects moderate colloidal stability in aqueous media, providing sufficient electrostatic repulsion to mitigate further aggregation despite the broad size distribution^55^ (SI Fig. 5).

**Fig. 5:**
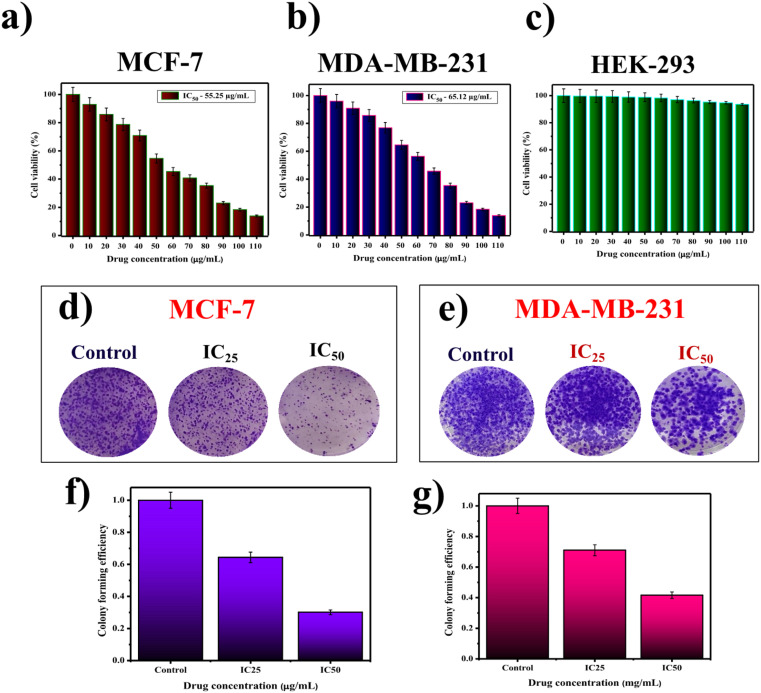
Cytotoxic and clonogenic analysis of SiQDs: (a–c) MTT assay results showing cell viability in MCF-7, MDA-MB-231, and HEK-293 cell lines; (d and e) clonogenic assay images illustrating colony formation in MCF-7 and MDA-MB-231 cells; (f and g) quantitative analysis of the percentage of colony-forming units (CFUs) in MCF-7 and MDA-MB-231 cells, respectively. The data are represented as mean ± standard deviation from three independent experiments.

Apart from the detailed physicochemical analysis, SiQDs were evaluated for anticancer properties against two intrinsic subtypes of human breast cancer (MCF-7 and MDA-MB-231). To ensure this, cell viability using the MTT assay was deployed to ensure the cytotoxic properties of SiQDs.^56^ Treatment with SiQDs at different concentrations (0–100 μg mL^−1^) exhibits anti-proliferative effects, with half-maximum inhibitory concentrations (IC_50_) of 55.25 μg mL^−1^ and 65.12 μg mL^−1^, respectively (Fig. 5a and b). Interestingly, SiQDs were observed to be biocompatible with normal human embryonic cells (HEK-293) at concentrations up to and beyond 200 μg mL^−1^^57^ (Fig. 5c). SI Table 3 presents a comprehensive cytotoxic effect of as-prepared SiQDs in relation to values reported in existing literature studies (SI Table 7). In our study, cisplatin was used as a positive control, which exhibited an IC_50_ value of 18.6 μg mL^−1^ in MCF-7 and 1.2 μg mL^−1^ in MDA-MB-231 breast cancer cells. From these results, the selective index of SiQDs was found to be 3.44 and 4.97 for MCF-7 and MDA-MB-231 breast cancer cells, as shown in the SI Table 8.^58^

In the meantime, growth and proliferation of cancer cells is a tightly coordinated process.^59,60^ Hence, the assessment of tumor growth is a critical factor in terms of delivering an effective anti-tumor agent.^61^ In the view of this, the long-term ability of SiQDs in hampering human breast cancer (MCF-7 and MDA-MB-231) was evaluated by clonogenic assay^62^ (Fig. 5d–g). The results from the study showed that SiQDs drastically reduce the colony-forming ability of MCF-7 and MDA-MB-231 cells in a dose-dependent manner as compared to untreated control cells.

**Fig. 6:**
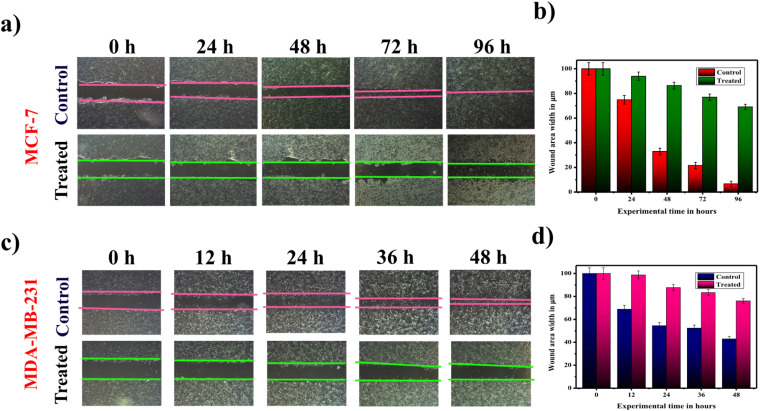
Scratch assay for wound healing and cell migration studies under control and treated (SiQDs) conditions. Representative images showing wound closure in (a) MCF-7 (0–96 h) and (c) MDA-MB-231 (0–48 h) cells at different time points. (b & d). Quantitative analysis of the percentage of wound healing in MCF-7 (b) and MDA-MB-231 (d) cells.

Besides, proliferation in tumors is accompanied by mobilization of cells.^63^ Therefore, a scratch or wound healing assay was performed using SiQDs.^64^ Since tumor cells have a typical property to “go and grow,” which is a unique feature of migration, correlated with the proliferation ability of these cells. In our study, untreated (control) cells exhibited rapid migration, leading to complete wound closure. With respect to increasing time, SiQDs treated breast cancer (MCF-7 and MDA-MB-231) cells depicted lower rates of migration as shown in (Fig. 6a–d).

Apart from the anti-proliferative properties of these QDs, these nanostructures exhibit potential anti-metastatic properties, one of the final attributes generated from the mobilization of cells. It was reported that, QDs conjugated with antibodies, deducing the expression of markers like epithelial cell adhesion matrix protein (EpCAM)/cluster of differentiation 44 (CD44), a key attributes involved in the process of epithelial-to-mesenchymal (EMT) or mesenchymal-to-epithelial (MET) transition, leading to enhanced migration, invasion, leading to metastasis.^65^

**Fig. 7:**
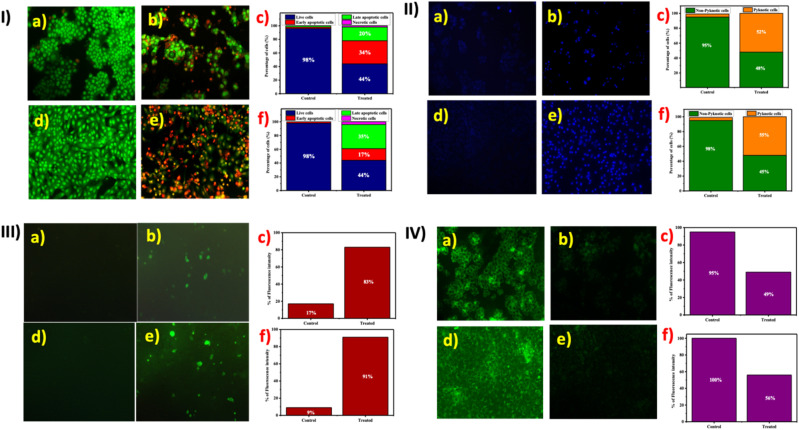
Fluorescence microscopic analysis of MCF-7 and MDA-MB-231 cells under control and treated (SiQDs) conditions: (I) dual staining (AO/EtBr) assay: (a & b) control cells; (d & e) treated cells; (c & f) quantification of apoptosis percentage in MCF-7 and MDA-MB-231 cells, respectively. (II) Hoechst 33342 staining: (a & b) control cells; (d & e) treated cells; (c & f) quantification of pyknotic cells with percentage in MCF-7 and MDA-MB-231 cells, respectively. (III) DCFH-DA staining: (a & b) control cells; (d & e) treated cells; (c & f) quantification of reactive oxygen species (ROS) generation in MCF-7 and MDA-MB-231 cells, respectively. (IV) Rhodamine-123 staining: (a & b) control cells; (d & e) treated cells; (c & f) quantification of mitochondrial membrane potential changes in MCF-7 and MDA-MB-231 cells, respectively.

Based on the anti-proliferative and anti-migratory properties of SiQDs against breast cancer cells, we were further interested in investigating the underlying mechanism *via* cellular imaging implementing various fluorescent dyes^66^ (Fig. 7). In most cases, anticancer agents are basically evaluated for the induction of programmed cell death (apoptosis). One of the gold standard techniques to affirm cell death at the morphological level in cancer cells is AO/EB, commonly referred to as dual staining.^67^

In dual-staining, live cells, or viable cells, are permeable to AO and emit green fluorescence, whereas dead cells, or non-viable cells, are impermeable to AO with compromised or disrupted membrane integrity and stain orange or red based on the stages of apoptosis.^68^ Here in this study, control cells stained green, inferring cell viability. To the contrary, SiQDs treated breast cancer cells at their respective IC_50_, revealing orange-red fluorescence, representing that SiQDs induced apoptosis in MCF-7 and MDA-MB-231 breast cancer cells (Fig. 7I-a–c). Based on the study, the dual-staining technique highlighted the different stages (*i.e.*, early apoptotic in yellow and late apoptosis in red) of inducing apoptosis at the cytoplasmic level (Fig. 7I-d–f).

Likewise, Hoechst staining was performed to assess apoptosis induction at the nuclear level upon treatment with SiQDs.^69^ Alteration of the nucleus is considered one of the key features for apoptosis. Hoechst 33342, a fluorescent stain that binds to minor grooves of A-T-rich regions of DNA, was employed to investigate the alterations induced at the nuclear level.^70^ Upon visualization under fluorescence microscopy, control cells depicted healthy nuclei, whereas SiQDs treated breast cancer cells portrayed condensed and fragmented nuclei, which are the key hallmarks of apoptosis induction at the nuclear level (Fig. 7II-a–c). As compared to the control cells, treated cells displayed a higher rate of apoptosis at both the cytoplasmic and nuclear levels (Fig. 7II-d–f).

In continuation with the above findings, wherein SiQDs restrained cancer cell growth and induced apoptosis, DCFH-DA staining was performed to gain further insights into the generation of reactive oxygen species (ROS) upon treatment with SiQDs^71^ (Fig. 7III-a–f). Interestingly, cells in the control displayed reduced green fluorescence, indicating a healthy and viable nature, whereas treated cells depicted a higher percentage of green fluorescence, causing an imbalance in cellular targets leading to mitochondrial perturbation in both breast cancer (MCF-7 and MDA-MB-231) cells^72^ (Fig. 7III).

QDs often trigger ROS-mediated cell death incells, where these nanodots act as photosensitizers and mediate energy transfer.^73,74^ For example, cadmium QDs generated ROS in HepG2 cells, which subsequently led to apoptosis.^75,76^ Earlier studies support that QDs trigger reactive oxygen (ROS)-mediated cell death in cancer cells, where these quantum materials act as a photosensitizers and mediate energy transfer.^77,78^ For instance, cadmium QDs generated ROS in prostate cancer cells, which subsequently led to apoptosis. In the same biosurfactant-stabilized cadmium QDs, induced free radical generation, thereby triggering oxidative stress leading to apoptosis.^79^

To examine if the induction of apoptosis was mitochondria mediated, we performed Rhodamine 123 stain, which is negatively charged and permeable to mitochondria, provided the cells retain intact morphology^80^ (Fig. 7IVa–f). In the present study, we found that SiQDs treated cells depicted a lower rate of green fluorescence as compared to control cells in both of the breast cancer (MCF-7 and MDA-MB-231) cells, indicating the fact that these SiQDs have the ability to alter the mitochondria membrane potential. In general conditions, normal cells don't exhibit overproduction of ROS, thereby suggesting that ROS plays a crucial role in stimulating apoptosis whereby mitochondrial membrane depolarization is involved, consequently leading to the generation of apoptotic molecules in the cytoplasm in cancer cells.^81–83^ In the same way, Luminous blue carbon QDs from *Anisomeles indica* induced mitochondrial-mediated cell death in triple-negative breast cancer (MDA-MB-231) cells.^31^

**Fig. 8:**
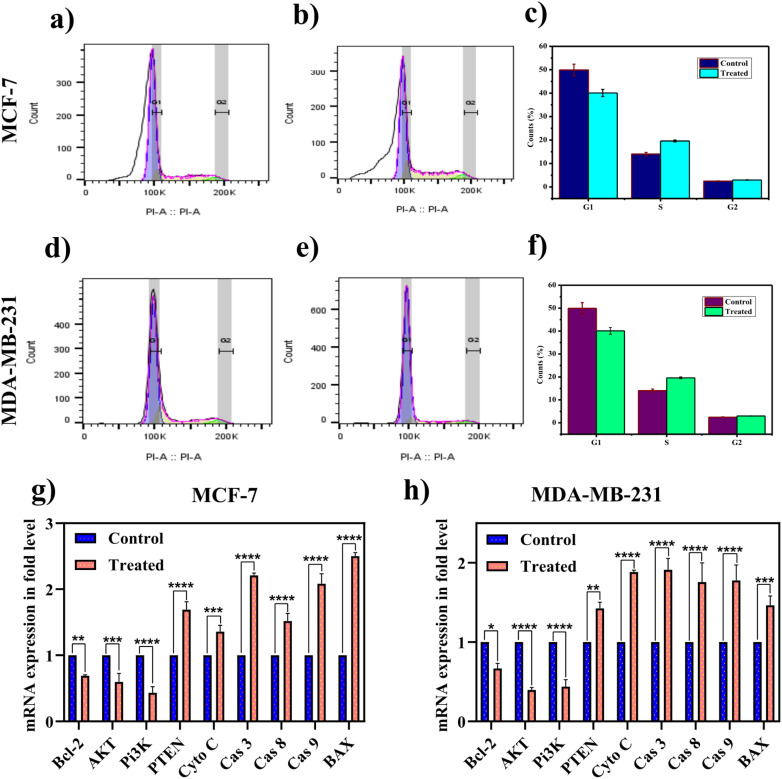
Cell cycle analysis of MCF-7 and MDA-MB-231 cells under control and silica quantum dot-treated conditions: representative histograms showing cell cycle distribution in MCF-7 (a & b) and MDA-MB-231 (d & e) cells; quantification of the percentage of cell cycle arrest in MCF-7 (d) and MDA-MB-231 (f) cells. Semi-quantitative RT-PCR analysis for gene expression studies in MCF-7 (g) and MDA-MB-231 (h) upon treatment with SiQDs. In our study, band intensity was quantified and normalized using an internal control (β-actin). The data are represented as mean ± standard deviation from three independent experiments.

Flow cytometry analysis of MCF-7 and MDA-MB-231 cells treated with SiQDs revealed distinct changes in cell cycle distribution^84^ (Fig. 8a–f). The observed effects of SiQDs on cell cycle dynamics reflect their potential as targeted therapeutic agents against breast cancer cells. The significant arrest in the S phase for MCF-7 cells suggests interference with DNA replication machinery, possibly due to the generation of oxidative stress or direct interaction with nuclear DNA (Fig. 8a–c). Conversely, the G2/M arrest in MDA-MB-231 cells could indicate disruption of mitotic spindle assembly or activation of checkpoint signaling pathways that prevent mitotic progression in response to DNA damage (Fig. 8d–f). In a study reported by Ku *et al.*, graphene QDs induced cytotoxicity *via* upregulated expression of p21 and p27, accompanied by enhanced levels of G2/M cell cycle arrest, inducing apoptosis in breast cancer cells.^85^

Based on the preliminary analysis, it is confirmed that SiQDs restrained the breast cancer growth, proliferation, and migration by inducing apoptosis in a dose- and time-dependent manner. Taking this into account, the molecular mechanisms underlying the signalling pathways associated with cell proliferation (PI3K/AKT/PTEN), mitochondrial regulation (Bax/Bcl-2/Cytochrome C), and apoptosis (Caspase-3, -8, and -9) were investigated using semi-quantitative gene expression analysis^35^ (SI, Fig. 6). These signaling molecules are crucial elements involved in regulating apoptosis *via* intrinsic or extrinsic pathways against several cancers, including breast (Fig. 8g and h).

In the meantime, PI3K (Phosphoinositide 3-kinase), a heterodimer primarily activated by receptor tyrosine kinase (RTKS) in response to growth factors.^86^ As a result, PI3K produces PIP3, which recruits kinases like PDK1 and AKT, initiating a cascade that regulates growth, proliferation, and related protein synthesis.^30^ Besides, phosphate and tensin homolog (PTEN), a key modulator that eventually downregulates the PI3K/AKT pathway with a synchronized upregulation that persuades apoptosis.^87^ As expected, treatment with SiQDs in breast cancer cells upregulates PTEN (a tumor suppressor gene) and turns down the PI3K/AKT signaling pathway (Fig. 8g and h).

In the meantime, the ratio of Bcl-2 and Bax within the mitochondria is crucial for cell survival.^88^ It has been noted that Bcl-2 expression downregulates Bax in cancer cells, serving a key turning point in terms of anti-tumor therapeutics^89^ (Fig. 8g and h). In our study, treatment with SiQDs led to the upregulation of Bax and the downregulation of Bcl-2, triggering the release of cytochrome c from mitochondria and initiating mitochondrial-mediated apoptosis^90^ (Fig. 8g and h). As a result, downstream caspases (cysteine aspartate proteases) persuade apoptosis to be a fundamental target of interest in the era of novel anticancer therapeutics.^91^

On the one hand, it has been reported that caspase-9 and -3 are involved in mitochondrial-mediated apoptosis^92^ (Fig. 8g and h). On the other hand, caspase-8 extrinsically activates caspase-3, a terminal factor leading to cell death.^93^ In the present study, SiQDs provoke the release of cytochrome c, which is accompanied by enhanced levels of caspase-9 and -3, indicating the fact that these QDs have the ability to trigger mitochondria, leading to the activation of the intrinsic pathway of apoptosis (Fig. 8g and h). It is also noted from the study that elevated expression of caspase-8 might trigger downstream caspases, thereby enhancing the chance of the extrinsic pathway of apoptosis^94,95^ (Fig. 8g and h).

Nguyen *et al.* reported that these cadmium telluride QDs induced the release of cytochrome C from mitochondria, with an enhancement of expression of Bax and subsequent downregulation of Bcl2.^96^ Studies reported that copper oxide Q dots (CuO) induced cytotoxicity and induced mitochondria-mediated cell death *via* enhanced expression of caspase-3 and caspase-7 in mouse C2C12 cells.^97^ Based on the above observation, it is clear that SiQDs have the ability to induce cytotoxicity, inhibit colony formation, growth, proliferation, and migration by triggering mitochondria-mediated apoptosis facilitated *via* downregulation of PI3K/AKT and Bcl-2 and concurrent up-regulated expression of caspase-3, 8, and 9, Bax and cytochrome-C in two intrinsic subtypes of breast cancer (MCF-7 and MDA-MB-231) cells.

## 4 Conclusion

To conclude, quantum-sized silica dots (SiQDs) were fabricated through microwave-assisted synthesis using TEOS and ascorbic acid, which exhibited dual functions as a reducing and surface passivating agent. The SiQDs showed strong, excitation-dependent fluorescence, absorbed UV-vis spectroscopy at 251 nm and FTIR spectra features indicative of formation and surface functionalization. As characterized by TEM and DLS, the SiQDs were primarily spherical and amorphous with a narrow size distribution peaking at ∼6.06 nm, featuring a moderate colloidal stability (zeta potential: −7.91 mV). Biologically, SiQDs showed selective anticancer activity against MCF-7 and MDA-MB-231 breast cancer cells, while maintaining biocompatible with normal HEK-293 cells. Functional assays showed that SiQDs inhibited cells' ability to proliferate, migrate and form colonies, while also inducing apoptosis through mitochondrial dysfunction and ROS generation. The molecular studies showed SiQDs modulated the PI3K/AKT signaling pathways to downregulate, while PTEN, Bax, cytochrome C, and caspases unregulated, demonstrating activation of both intrinsic and extrinsic apoptotic pathways. These results propose SiQDs as a therapeutic nanoplatform with improved biocompatibility, showcasing their promise in cancer therapy.

## Ethical statement

No live animals or human participants were involved in this study. The cell lines used are established and do not require ethical approval for use.

## Author contributions

S. A. performed all experiments under the mentorship of P. K. P. K. wrote and critically reviewed the manuscript. P. K. acquired funding to execute this project.

## Conflicts of interest

The authors declare no competing interest.

## Supplementary Material

RA-015-D5RA04715C-s001

RA-015-D5RA04715C-s002

## Data Availability

The datasets used/analyzed in the study are available from the corresponding author based on a reasonable request. Supplementary information: additional experimental data, characterization results, and supporting figures referenced in the main text. See DOI: https://doi.org/10.1039/d5ra04715c.
