# Supplementary figures and images for: Microwave-assisted synthesis of silica quantum dots: a novel approach for targeting PI3K/AKT signaling in breast cancer therapy
Source: RSC Adv. 2025 Oct 20;15(47):39677–88. doi: 10.1039/d5ra04715c (PMC12536649; doi:10.1039/d5ra04715c)

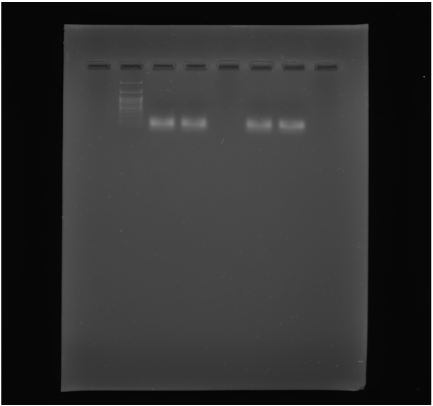

Supplement: RA-015-D5RA04715C-s001 [file RA-015-D5RA04715C-s001.zip › MCF-Actin.tiff]

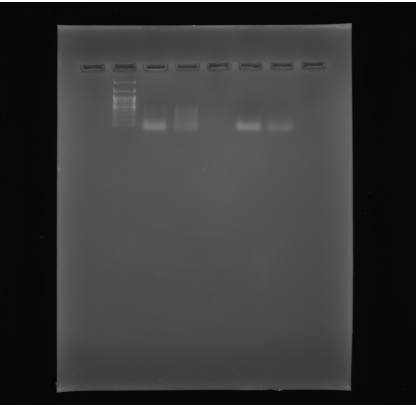

Supplement: RA-015-D5RA04715C-s001 [file RA-015-D5RA04715C-s001.zip › MCF-AKT.tiff]

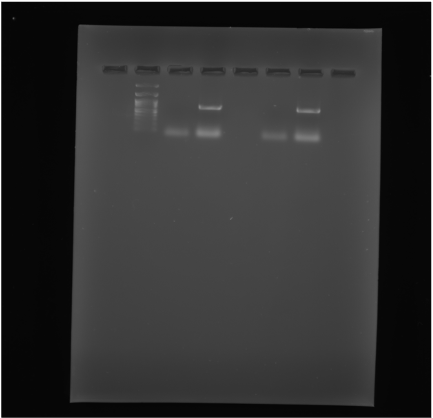

Supplement: RA-015-D5RA04715C-s001 [file RA-015-D5RA04715C-s001.zip › MCF-BAX.tiff]

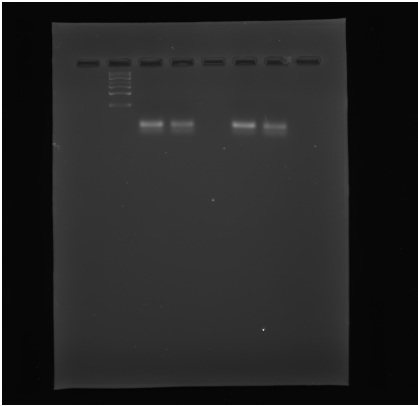

Supplement: RA-015-D5RA04715C-s001 [file RA-015-D5RA04715C-s001.zip › MCF-BCL2.tiff]

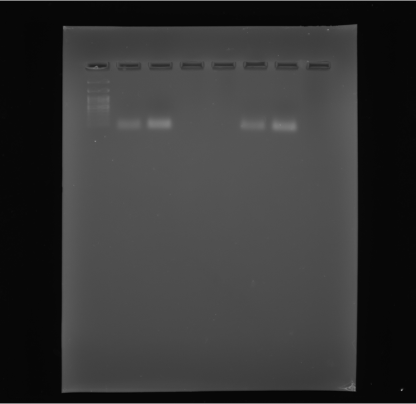

Supplement: RA-015-D5RA04715C-s001 [file RA-015-D5RA04715C-s001.zip › MCF-CAS-3.tiff]

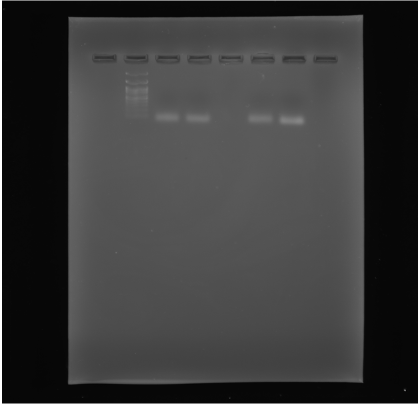

Supplement: RA-015-D5RA04715C-s001 [file RA-015-D5RA04715C-s001.zip › MCF-CAS-8.tiff]

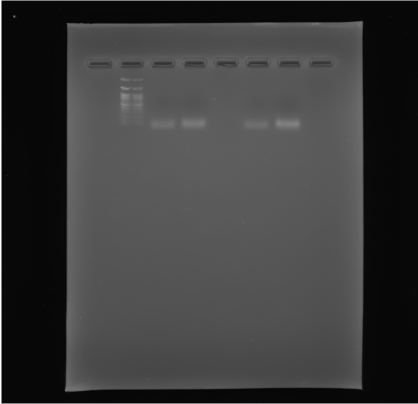

Supplement: RA-015-D5RA04715C-s001 [file RA-015-D5RA04715C-s001.zip › MCF-CAS-9.tiff]

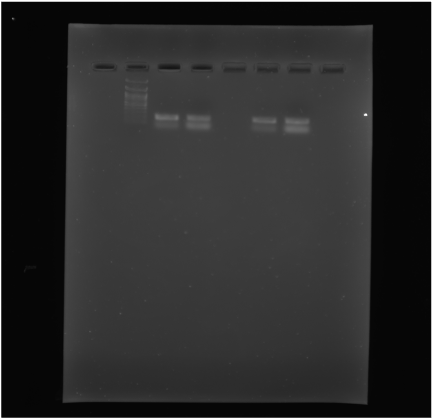

Supplement: RA-015-D5RA04715C-s001 [file RA-015-D5RA04715C-s001.zip › MCF-CYTO.tiff]

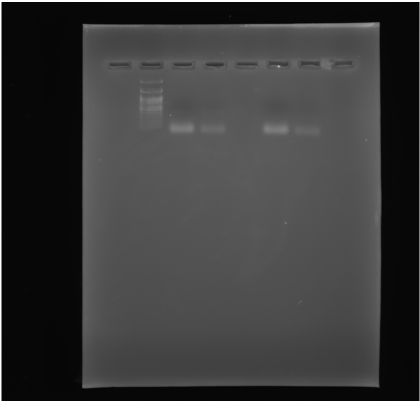

Supplement: RA-015-D5RA04715C-s001 [file RA-015-D5RA04715C-s001.zip › MCF-PI3K.tiff]

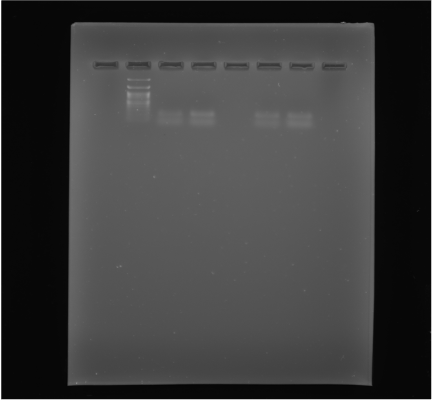

Supplement: RA-015-D5RA04715C-s001 [file RA-015-D5RA04715C-s001.zip › MCF-PTEN.tiff]

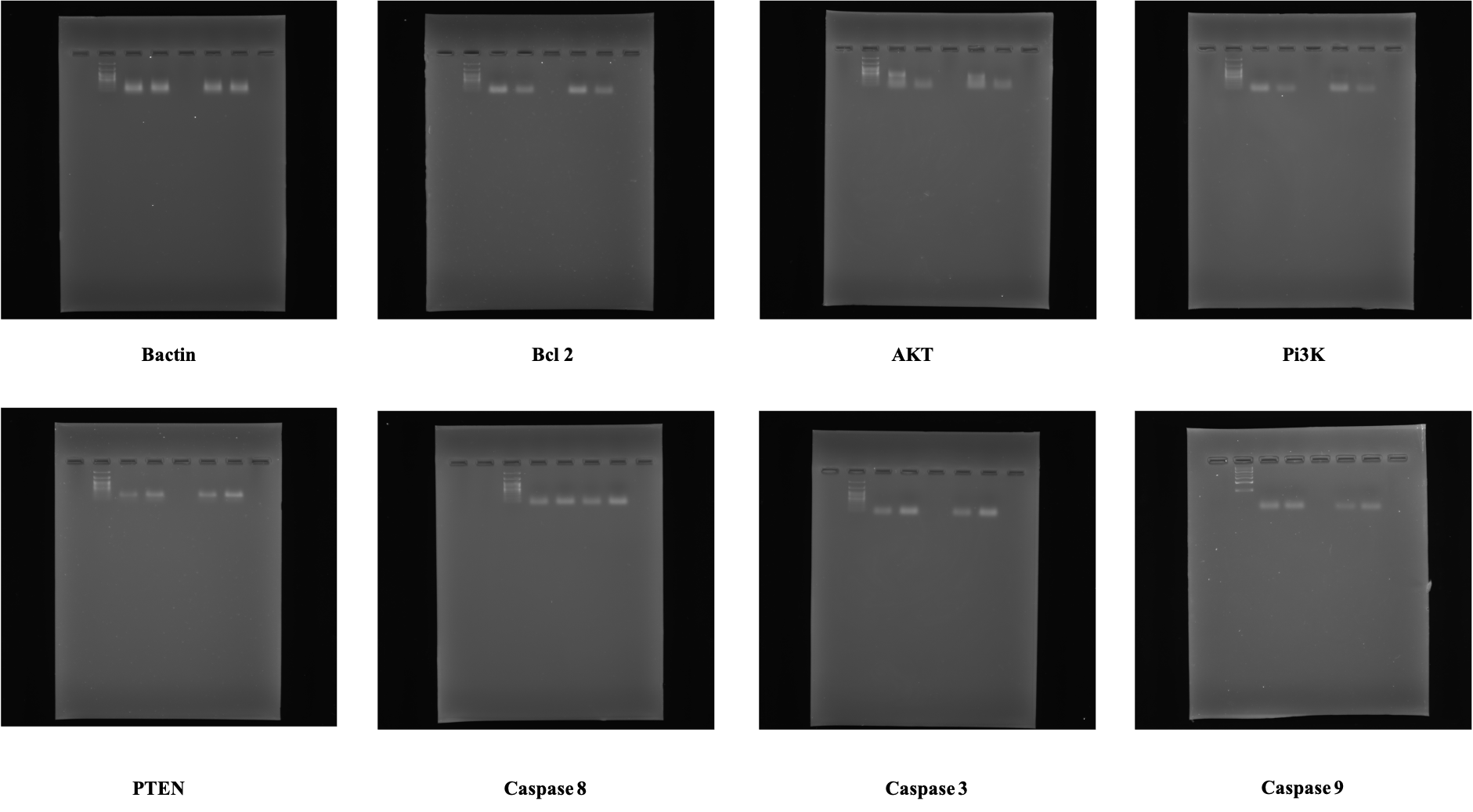

Supplement: RA-015-D5RA04715C-s001 [file RA-015-D5RA04715C-s001.zip › MDA-ACTIN.tiff]

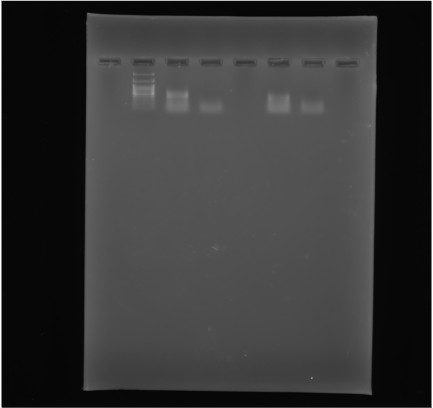

Supplement: RA-015-D5RA04715C-s001 [file RA-015-D5RA04715C-s001.zip › MDA-AKT.tiff]

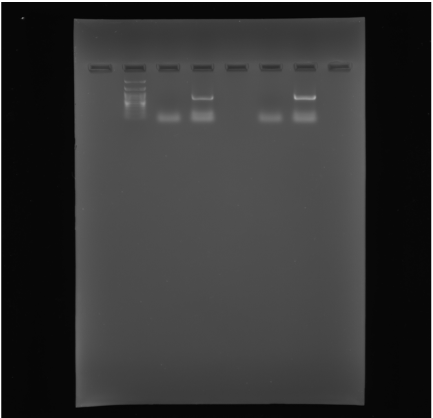

Supplement: RA-015-D5RA04715C-s001 [file RA-015-D5RA04715C-s001.zip › MDA-BAX.tiff]

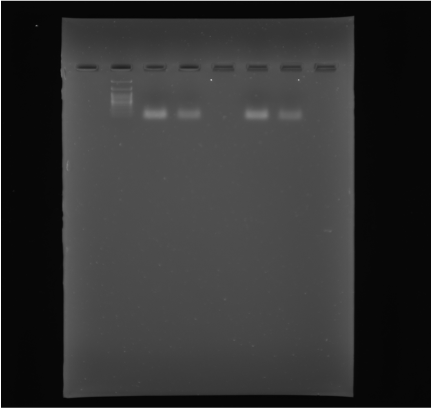

Supplement: RA-015-D5RA04715C-s001 [file RA-015-D5RA04715C-s001.zip › MDA-BCL2.tiff]

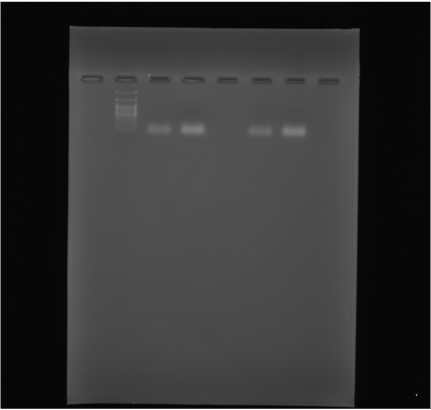

Supplement: RA-015-D5RA04715C-s001 [file RA-015-D5RA04715C-s001.zip › MDA-CAS-3.tiff]

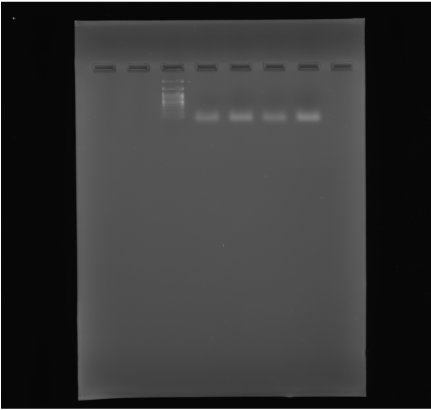

Supplement: RA-015-D5RA04715C-s001 [file RA-015-D5RA04715C-s001.zip › MDA-CAS-8.tiff]

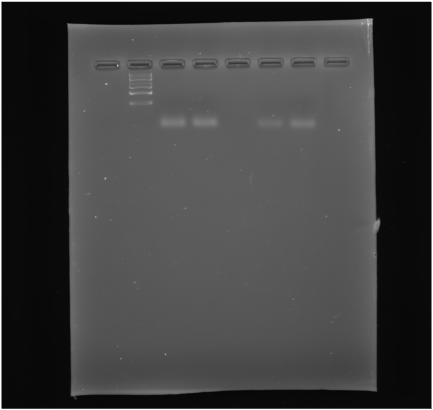

Supplement: RA-015-D5RA04715C-s001 [file RA-015-D5RA04715C-s001.zip › MDA-CAS-9.tiff]

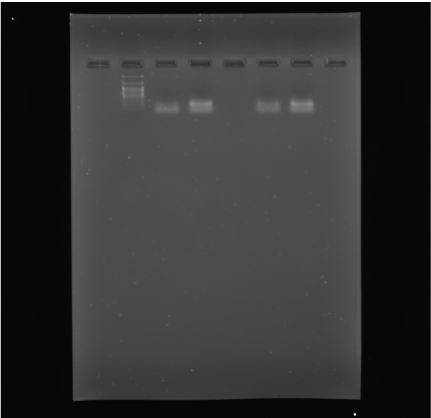

Supplement: RA-015-D5RA04715C-s001 [file RA-015-D5RA04715C-s001.zip › MDA-CYTO.tiff]

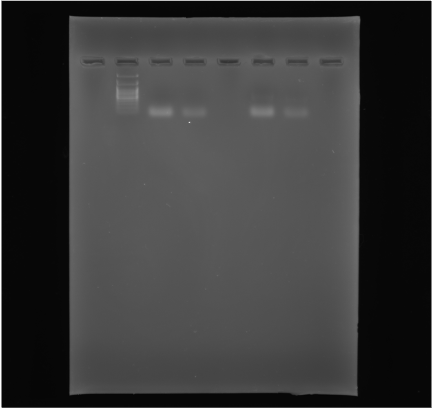

Supplement: RA-015-D5RA04715C-s001 [file RA-015-D5RA04715C-s001.zip › MDA-PI3K.tiff]

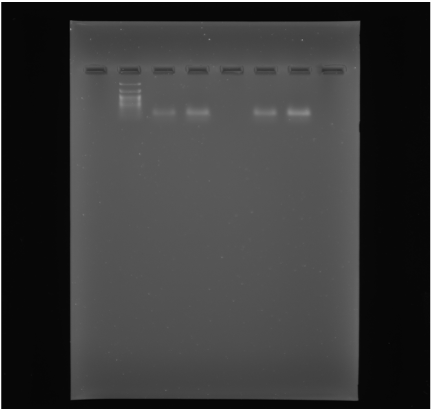

Supplement: RA-015-D5RA04715C-s001 [file RA-015-D5RA04715C-s001.zip › MDA-PTEN.tiff]
