# Supplementary material for: Microwave-assisted synthesis of silica quantum dots: a novel approach for targeting PI3K/AKT signaling in breast cancer therapy
Source: RSC Adv. 2025 Oct 20;15(47):39677–88. doi: 10.1039/d5ra04715c (PMC12536649; doi:10.1039/d5ra04715c)
Supplement: RA-015-D5RA04715C-s002 [file RA-015-D5RA04715C-s002.pdf]

Supplementary Information (SI) for RSC Advances.
This journal is © The Royal Society of Chemistry 2025

SUPPLEMENTARY INFORMATION

Microwave-Assisted Synthesis of Silica Quantum Dots: A Novel Approach for Targeting PI3K/AKT Signaling in Breast Cancer Therapy

Selvaraj Anitha and Ponnuchamy Kumar*

Food Chemistry and Cancer Biology Lab, Department of Animal Health and Management, Alagappa University, Karaikudi – 630 003, Tamil Nadu, India

*** Corresponding author**

Dr. Ponnuchamy Kumar – kumarp@alagappauniversity.ac.in

SI Table. 1 Primers used

Apoptotic Gene	Primers used
AKT	F - 5’- CAGTGGACCACCTTCGTTGA - 3’<br>R - 5'- ACAGAGTCGGCCACTGATTG - 3'
PI3K	F - 5’- GGAAGCCCTCCAGAAAGGTC - 3’<br>R - 5’- GCACTCGGAAGTTGAATGGC - 3’
PTEN	F - 5'- TCCCAGACATGACAGCCATC - 3'<br>R - 5’- TGTCTTTCAGCACAACTTACTACA - 3’
BAX	F - 5’- AGCAAACTGGTGCTCAAGGC - 3’<br>R - 5’- CAGGGACATCAGTCGCTTCAG - 3’
Bcl-2	F - 5’- F-CTTTGAGTTCGGTGGGGTCA - 3’<br>R - 5’- GGGCCGTACAGTTCCACAAA - 3’
Cytochrome c	F - 5’- ACAAAGGCATCATCTGGGGAG - 3’<br>R - 5’- AGGCAGTGGCCAATTATTACTC - 3’
Caspase-9	F - 5'- TGAGACCCTGGACGACATCT - 3'<br>R - 5’- TCCCTTTCACCGAAACAGCA - 3’
Caspase-8	F - 5'- GCGGAGGGTCGATCATCTAT - 3'<br>R - 5'- TCCTTCTCCCAGGATGACCC - 3'
Caspase-3	F - 5’- GTGCTATTGTGAGGCGGTTG - 3’<br>R - 5’- TCCAGAGTCCATTGATTCGCTT - 3’
β-actin	F – 5’ – TGGAACGGTGAAGGTGACAG - 3’<br>R – 5’ – AACAACGCATCTCATATTTGGAA - 3’

SI Table. 2 Excitation-dependent Emission

S.No.	λ_{Ex} (nm)	λ_{Em}(nm)
1.	300	7511
2.	320	8786
3.	340	7279
4.	360	3678
5.	380	2436
6.	400	1389
7.	420	757
8.	440	302
9.	460	235
10.	480	141
11.	500	72
12.	520	43
13.	540	22
14.	560	10
15.	580	5

SI Table. 3 Functional groups present in Ascorbic acid via FTIR analysis

S. No.	Peak (cm^{-1})	Functional Group	Structural Origin
1.	3524, 3407, 3313	O–H stretching	Alcoholic/Phenolic hydroxyl groups
2.	1751	C=O stretching	Lactone ring/carboxylic group
3.	1653	C=C stretching	Conjugated double bonds/ring
4.	1311	C–O stretching	Carboxylic groups
5.	1111, 1017	C–O stretching	Alcoholic/ether linkages
6.	818, 750	C–H/O–H bending	Ring/fingerprint region vibrations

SI Table. 4 Functional groups present in TEOS via FTIR analysis

S. No.	Peak (cm^{-1})	Functional Group	Structural Origin
1.	2981, 2975	C–H stretching	Ethoxy CH_2, CH_3
2.	1390, 1295	C–H bending	CH_2, CH_3 groups
3.	1165, 1070	Si–O–C stretching	Ethoxy-Si linkage
4.	957	Si–OH stretching	Si–OH formation/hydrolysis
5.	785	Si–O stretching	Orthosilicate, Si framework

SI Table. 5 Functional groups present in SiQDs via FTIR analysis

S.No.	Peak (cm^{-1})	Assignment	Structural Origin
1.	3508	O–H stretching	Surface silanol, adsorbed water
2.	1640	H–O–H bending	Adsorbed water, residual C=C
3.	1085	Si–O–Si stretching	Siloxane network core
4.	948	Si–OH stretching	Surface uncondensed silanols
5.	794	Symmetric Si–O–Si	Silica framework

SI Table. 6 Structural Features of Chemical Compounds During SiQD Formation

S.No	Structural feature	Ascorbic acid	TEOS	SiQDs
1.	O-H stretch	Present (3313–3524 cm^{-1})	None	Present (3508 cm^{-1})
2.	C=O stretch	Present (1751 cm^{-1})	Absent	Absent
3.	Si–O–C	Absent	Present (1165–1070 cm^{-1})	Converted to Si–O–Si (1085 cm^{-1})
4.	Si–O–Si	Absent	Weak (785 cm^{-1})	Strong (1085, 794 cm^{-1})

SI Table. 7 Comparative literature on silica quantum dots

S. No.	Nanomaterial used	Methodology	Precursor agent	Reducing agent	Cell lines used	Reference
1	Nano-SiO2	-	-	-	MDA-MB-231 and HS578T - ~100 µg/ml	[1]
2	Galactose functionalized silica nanoparticles	Stobers method	TEOS	-	HUH-7 (2.5 mg/mL)	[2]
3	Phosphonate-Functionalized Mesoporous Silica Nanoparticles	Micelle-templating method	TEOS	-	MCF-7(IC-50 -250 µg/ml) BJ Cells (IC-70 -250 µg/ml)	[3]
4.	Sialic acid-targeting multi-functional silicon quantum dots	Bottom-up wet-chemical reduction	Silicon	Sodium ascorbate	MCF-7 – 6.4 µg/ml	[4]
5	Kaolin phyllosilicate-derived silica quantum dots	Acid-base hydrothermal method	Kaolin (Phyllosilicate)		B16F0 – 134 ppm and MCF-7 cells – 147 ppm.	[5]
6	Silica quantum dots (SiQDs)	Microwave irradiation method	TEOS	Ascorbic acid	MCF-7 - 58.12 µg/mL; MDA-MB-231 - 40.28 µg/mL and HEK-293 (beyond 200 µg/mL) cells	Present study

SI Figure 8 Selective index of SiQDs

S. No	Cell Line used	IC_{50} (Cancer cells, µg/mL)	IC_{50} (Normal HEK-293, µg/mL)	Selective index
1.	MCF-7	55.25	≥ 200	~ 3.61*
2.	MDA-MB-231	65.12	≥ 200	~ 3.07*

***SI > 1**: Indicates some selectivity — the compound affects cancer cells more than normal cells.

****SI = 1**, the compound is about equally toxic to both cancer and normal cells — poor selectivity.

*****SI < 1**, this suggests the compound might be more toxic to normal cells than to cancer cells — undesirable.

SI Figure. 1 – FTIR analysis of Ascorbic acid

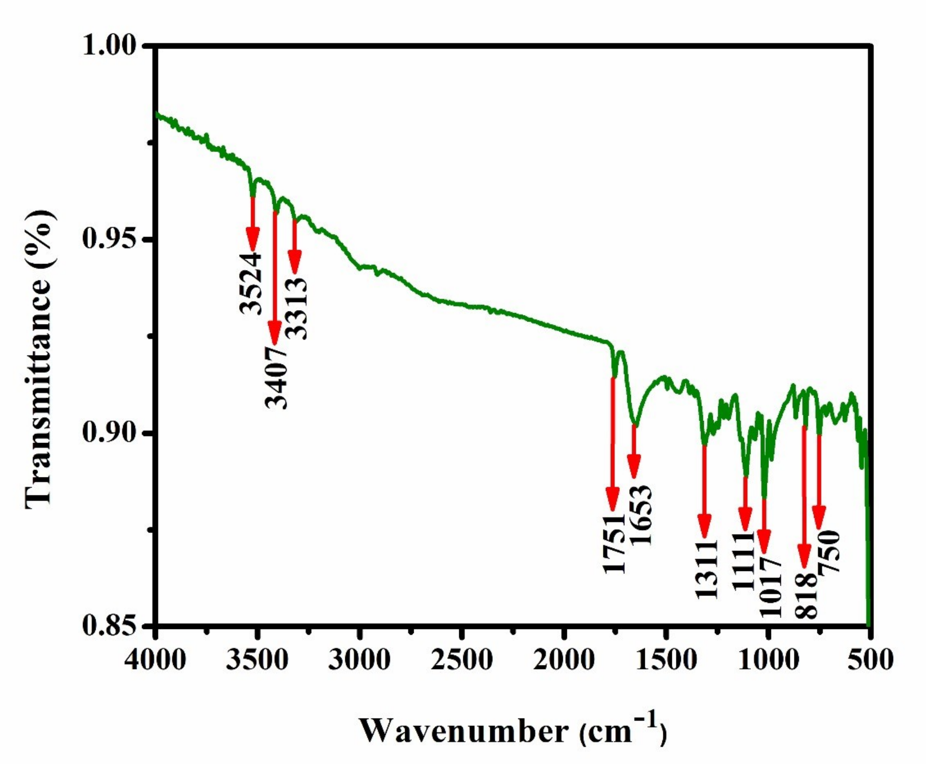

SI Figure. 2 – FTIR analysis of TEOS

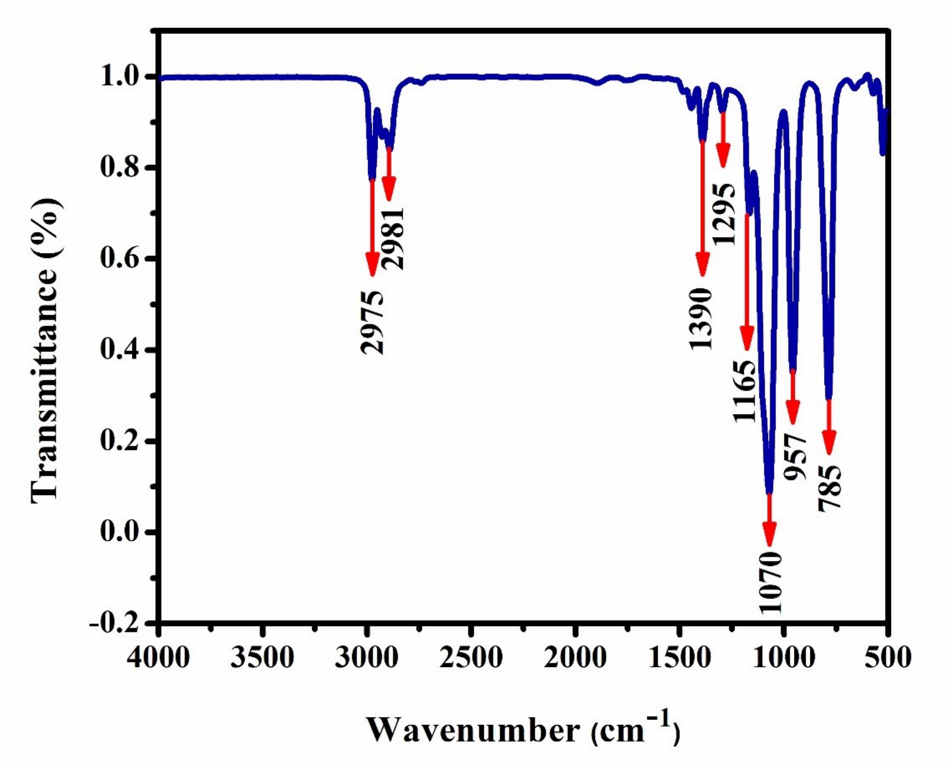

SI Figure. 3 – Average Particle Size of SiQDS

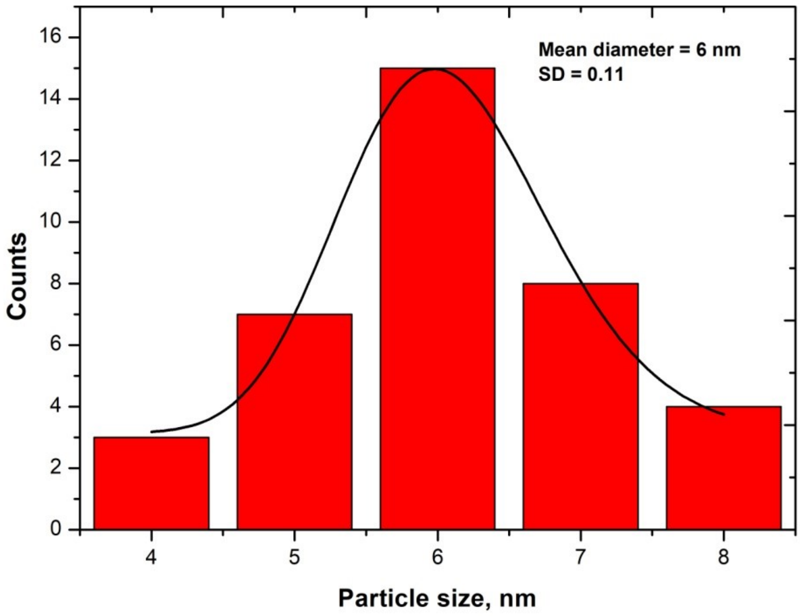

SI Figure. 4 Hydrodynamic diameter of SiQDs

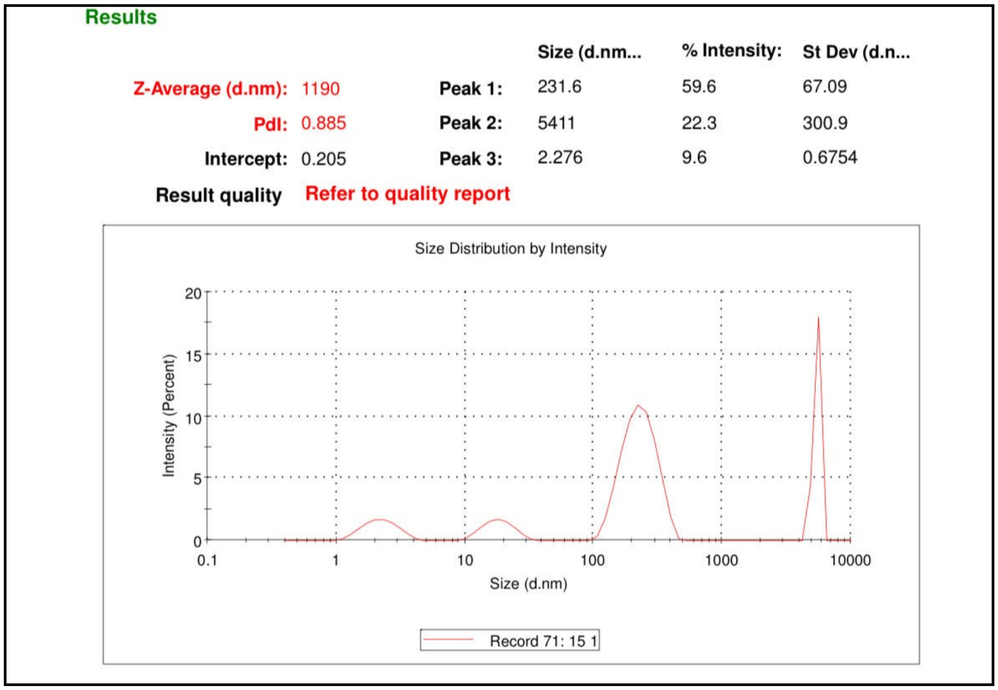

SI Figure. 5 Zeta potential measurement

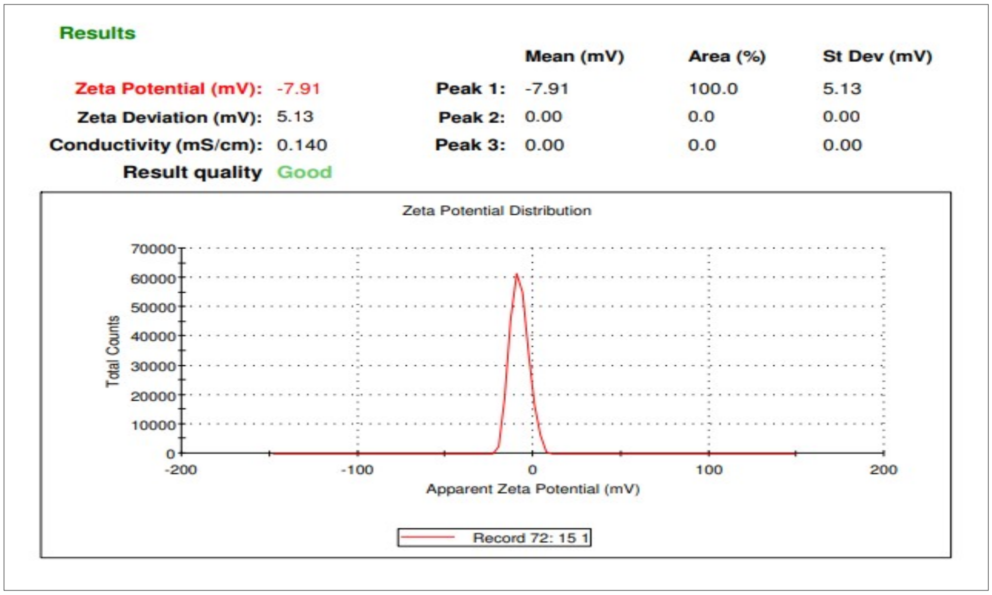

SI Figure. 6 Gene expression studies (Semi-quantitative RT-PCR analysis)

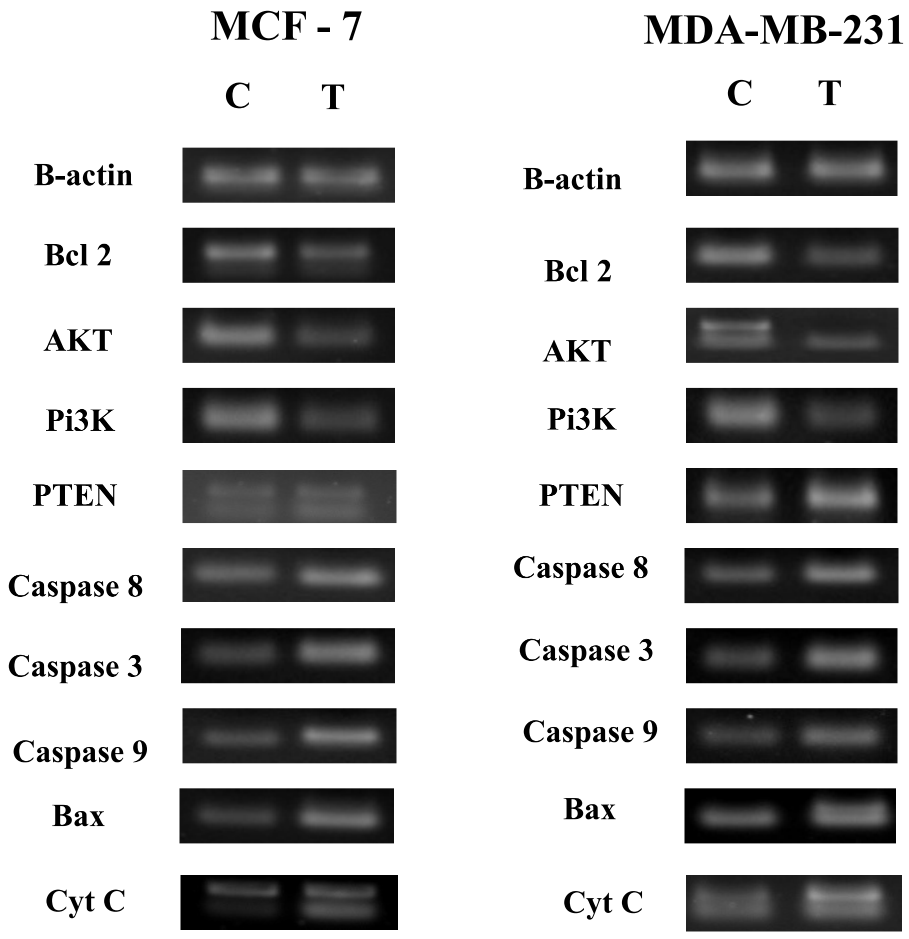

References:

[1] Jeon, D., Kim, H., Nam, K., Oh, S., Son, S., & Shin, I. (2017). Cytotoxic effect of Nano-SIO2 in human breast cancer cells via modulation of EGFR signaling cascades. *Anticancer Research*, *37*(11).

[2] Mukherjee, M. B., Mullick, R., Reddy, B. U., Das, S., & Raichur, A. M. (2020). Galactose functionalized mesoporous silica nanoparticles as delivery vehicle in the treatment of hepatitis C infection. *ACS Applied Bio Materials*, *3*(11), 7598–7610.

[3] Menon, N., & Leong, D. T. (2016). Cytotoxic effects of Phosphonate-Functionalized mesoporous silica nanoparticles. *ACS Applied Materials & Interfaces*, *8*(3), 2416–2422.

[4] Liu, F., Lin, J., Luo, Y., Xie, D., Bian, J., Liu, X., & Yue, J. (2023). Sialic acid-targeting multi-functionalized silicon quantum dots for synergistic photodynamic and photothermal cancer therapy. *Biomaterials Science*, *11*(11), 4009–4021.

[5] Irmania, N., Solihin, N., & Rosyidah, A. (2025). Kaolin phyllosilicate-derived silica quantum dots via acid-base hydrothermal synthesis for anti-cancer applications. *Minerals Engineering*, *233*, 109603.
